# Enhancement in the Detection Ability of Metal Oxide Sensors Using Defect‐Rich Polycrystalline Nanofiber Devices

**DOI:** 10.1002/gch2.202000041

**Published:** 2020-09-28

**Authors:** Chun‐Yen Lai, Yu‐Ting Lin, Hung‐Kun Hsu, Ding‐Yeong Wang, Wen‐Wei Wu, Ping‐Hung Yeh

**Affiliations:** ^1^ Department of Materials Science and Engineering National Chiao Tung University No. 1001, University Road, East District Hsinchu City 30010 Taiwan; ^2^ Department of Physics Tamkang University No. 151, Yingzhuan Road, Tamsui District New Taipei City 25137 Taiwan; ^3^ Electronic and Optoelectronic System Research Laboratories Industrial Technology Research Institute Hsinchu 31040 Taiwan; ^4^ Material and Chemical Research Laboratories Nanotechnology Research Center Industrial Technology Research Institute Hsinchu 310 Taiwan; ^5^ Frontier Research Center on Fundamental and Applied Sciences of Matters National Tsing Hua University Hsinchu City 300 Taiwan

**Keywords:** interface defects, nanofibers, photon‐sensing devices, SnO_2_, toxic gasses sensors

## Abstract

The development of SnO_2_ and TiO_2_ polycrystalline nanofiber devices (PNFDs) has been widely researched as a method of protecting humans from household air pollution. PNFDs have three significant advantages. The nanofibers before the annealing process are polymer‐rich materials, which can be used as particulate material (PM) filters. The multiporous nanofibers fabricated by the annealing process have numerous defects that can serve as generation‐recombination centers for electron–hole pairs, enabling the PNFDs to serve as multiple‐wavelength light (from 365 to 940 nm) detectors. Lastly, the numerous surface/interface defects can drastically enhance the toxic gas detection ability. The toxic gas detection range of PNFDs for CO_(g)_ and NO_(g)_ is from 400 to 50 ppm and 400 to 50 ppb, respectively. Quick response times and recovery properties are key parameters for commercial applications. The recovery time of NO_(g)_ detection can be improved from 1 ks to 40 s and the PNFD operating temperature lowered to 50 **°**C. These results indicate that SnO_2_ and TiO_2_ PNFDs have good potential for commercialization and use as toxic gas and photon sensors in daily lives.

## Graphical abstract

Defect‐rich SnO_2_ and TiO_2_ polycrystalline nanofiber devices (PNFDs) are fabricated and tested. These materials have strong electrostatic force properties, ultrafast detection abilities at multiple wavelengths (365–940 nm), and toxic gas‐sensing abilities. Therefore, PNFDs can be used as particulate material filters, multiple‐wavelength light detectors, and next‐generation commercial household appliances to monitor indoor air pollution at room temperature.

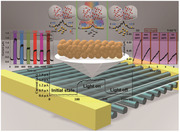

Over the past few decades, ambient air pollution (AAP) sources, including exhaust fumes, toxic air, and particulate materials (PMs), have increased due to power generation, vehicle emissions, and the gas industry. This has compromised environmental safety. In 2018, the World Health Organization (WHO) reported the effects of AAP and household air pollution (HAP) on human health. According to these reports, outdoor air pollution has not only gradually changed our living habits, but also increased the probability of death and diseases, including lung cancer, strokes, ischemic heart disease, and acute respiratory infections. Another report from the WHO in 2016 mentioned that AAP and HAP had caused the death of 543 000 children under the age of 5 years.^[^ ^1^, ^2^ ^]^ These reports indicate a direct link between AAP, HAP, and human health. HAP includes volatile organic compounds, PMs, CO, SO_2_, and NO. Cooking, heating, and burning solid or liquid fuels such as wood, coal, kerosene, and gasoline generate large amounts of pollutants, which strongly impact human health, especially that of young children and people who spend majority of their time indoors. According to the Occupational Safety and Health Administration (OSHA) regulations of the United States Department of Labor, the concentrations of CO and NO gas in a workplace should not exceed 35–50 ppm and 30–45 ppm, respectively. CO and NO, which are colorless, tasteless, and have nonirritant properties,^[^ ^3^, ^4^, ^5^, ^6^, ^7^ ^]^ are toxic gases. If people are exposed to a CO and NO gas environment for a long time, these gases dissociate into their blood and combine with hemoglobin (Hb) to reduce the oxygen‐carrying capacity of the blood. Hence, it is important to prevent or minimize HAP. To reduce AAP and HAP (CO, SO_2_,and NO*_x_*), semiconductor materials (ZnO, TiO_2_, and SnO_2_) could be converted into high porosity polycrystalline nanofibers (PNFs), which have many advantages, including a nanointerface between nanograins,^[^ ^8^, ^9^ ^]^ wide band gap (3.6 eV), and generation‐recombination centers, owing to the defects and dangling bonds at the end of nanograin's surface. Recent studies on the development and application of PNFs suggest that they can be used in photonic devices, such as photon sensors^[^ ^10^, ^11^, ^12^, ^13^ ^]^ and solar cells,^[^ ^14^, ^15^ ^]^ as well as non‐photonic devices, such as PM filters,^[^ ^16^, ^17^ ^]^ toxic gas sensors,^[^ ^18^, ^19^, ^20^, ^21^, ^22^, ^23^, ^24^, ^25^, ^26^, ^27^, ^28^, ^29^, ^30^, ^31^, ^32^, ^33^, ^34^ ^]^ biological sensors,^[^ ^35^, ^36^, ^37^, ^38^, ^39^ ^]^ RRAM devices,^[^ ^40^, ^41^ ^]^ and energy devices.^[^ ^42^, ^43^, ^44^, ^45^, ^46^, ^47^, ^48^, ^49^ ^]^ The indoor ambient temperature for the majority of households is ≈25–30 °C. According to Yang and co‐workers^[^ ^50^ ^]^ and Moon et al.,^[^ ^29^ ^]^ SnO_2_ and TiO_2_ nanofiber devices are used to enhance H_2_ and NO_2_ gas sensitivity. A change in the grain boundary by Pd doping in the precursors could improve the results achieved by these devices. However, even if the gas sensitivity can be enhanced, the nanofiber devices will need to be operated at high temperatures (≈180–380 °C). Furthermore, according to Kim et al.,^[^ ^24^ ^]^ SnO_2_ nanofiber devices tend to increase the CO gas detection concentration at the parts per million level by Au functionalization. Even though these devices have good detection ability, they still need to be operated at a high temperature (≈400 °C). In summary, SnO_2_ and TiO_2_ nanofiber devices can only be operated at high temperatures and require doping with precious metals as well. Therefore, PNFDs could be more powerful and useful if operated at lower temperatures and without precious‐metal doping.

In our previous studies, we fabricated nanointerface defects, defect engineering,^[^ ^51^ ^]^ and surface defects,^[^ ^52^ ^]^ which enhanced the gas sensing and photon detection abilities of PNFDs. Using surface engineering, a single crystal metal oxide nanowire device with various defect densities and different surface oxygen vacancies (Vo) can be formed on a nanostructure. The detection increases as the surface defect density increases. For interface defect engineering, nanointerface defects create various defect energy levels that lead to multiple‐wavelength light detection. The current flow past the interface can generate heat (Joule heating effect). Local heating can enhance gas absorption and improve gas detection ability while lowering the operating temperature of detectors. In this work, PNFDs were designed with both surface and interface defects for multifunction ability, based on the aforementioned ideas.

To create nanostructures with surface and interface defects, defect‐rich PNFs were fabricated by the electron‐spinning process; a schematic illustration is shown in **Figure** **1**a. A PNFD was formed using multiple nanofibers as well as a single nanofiber, as shown in Figure 1b. The results of the transmission electron microscopy (TEM) analysis of the polycrystalline structure are shown in Figure 1c,d. From the low‐magnification TEM images, it can be seen that the grain sizes are uniform (≈11 nm) and SnO_2_ nanofibers were smaller compared to TiO_2_ nanofibers (≈38 nm). High‐resolution TEM and diffraction pattern images revealed that the nanofibers had plenty of grain boundaries between nanograins with different crystal planes, where the interface defect exists. The diffraction patterns of the SnO_2_ and TiO_2_ nanofibers also demonstrated the randomness of the PNFs.

**Figure 1:**
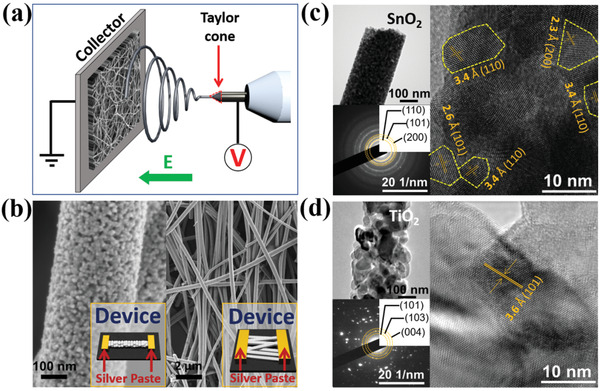
Schematic illustration of the electrospinning process and electronic microscope analysis. a) Nanofiber fabrication by the electrospinning process. b) SEM image of the SnO_2_‐based nanodevice fabricated with single or multiple nanofibers. c,d) Low‐magnification, high‐resolution, and diffraction pattern of SnO_2_ and TiO_2_ nanofibers obtained by TEM analysis, respectively.

To check the properties of the PNFs, multiple‐wavelength light can be used to prove the existence of interface and surface defects. SnO_2_ (3.6 eV) and TiO_2_ (3.2 eV) are the two metal oxides with wide band gaps that can be used to demonstrate the structural properties of the nanointerface defects. In previous studies, pure SnO_2_ nanofiber photodetectors indicated single narrow wavelength (450 nm) photodetection. Some articles mentioned that the visible light detection abilities of metal oxide nanofibers (SnO_2_ and TiO_2_) could be achieved by chemical functionalization, metal element doping, and nanocomposite heterojunction interfaces^[^ ^51^ ^]^ between two or more semiconductor materials. In this study, the defect‐rich SnO_2_ PNFD was formed with numerous defects. The defect‐rich SnO_2_ PNFDs are used as multiple‐wavelength (from 365 to 940 nm) photodetectors. The numerous defects of the nanograin interfaces can be used as generation‐recombination centers that react with the multiple‐wavelength light, as illustrated in Figure 1c. Multiple‐wavelength light sources (from 365 to 940 nm) were used as the photo source in this study. The photo‐sensing ability and photo‐dark current variations under different wavelengths of light are shown in **Figure** **2**. The photoresponse, sensitivity *S* (red arrow), and current variation Δ*I* (black arrow) of the SnO_2_ PNFD are shown in Figure 2a,b. A single‐fiber SnO_2_ PNFD was also fabricated to show the detection result, as illustrated in Figure S1, Supporting Information. The photocurrent response decreases as the wavelength of light increases (from 365 to 940 nm) because long‐wavelength light can only generate electron–hole pairs from low‐energy level defects. The defect energy levels of PNFDs should be different, so that low energy light can generate less electron–hole pairs and have low current outputs. With the same polycrystalline structure, other metal oxide materials, such as TiO_2_, can perform similar multiple‐wavelength photodetection to SnO_2_ PNFDs, as shown in Figure 2c,d. To create more defects and increase the output current of the TiO_2_ PNFD, hydrogen plasma treatments were explored (40, 80, and 120 W). The current output can be enhanced from 7 nA (without) to 335 nA with hydrogen plasma treatments at 120 W in 30 s, as shown in Figure S2, Supporting Information. However, the current output of the SnO_2_ PNFDS is considered better than that of the TiO_2_ PNFD. The multiple‐wavelength light‐sensing mechanisms in PNFDs are attributed to two different photoge nerated electron effects. One is the movement of photogenerated free electrons from the valence band to the conduction band (direct band gap 3.6 eV). The other is the generation of photoexcited electrons from the generation‐recombination centers owing to the random defect energy level states at the interfaces of the grains. Three steps are proposed to explain the photodetection process in the defect‐rich structure of nanograins, as shown in **Figure** **3**. State I (initial state) has low current output and the SnO_2_ nanofiber has various grain boundaries and interfaces that have numerous defects that trap the free electrons and lead to low initial dark current (≈0.6 µA). State II (illumination state) has high photocurrent output, and the defects between the grain interfaces serve as generation states to generate photocurrent (≈1.5 µA) under various wavelengths (from 365 to 940 nm) of light illumination. The defect states between the grain interfaces are abundant and randomly located. Thus, the trapped electrons in different defect energy levels can be excited and generated from various current output levels by different wavelengths of light illumination. The photocurrent response decreases when the wavelength increases owing to the smaller energy of long‐wavelength light. State III (non‐illumination state) has decreasing current output and the various defects of the grain interfaces act as recombination centers of free electrons with holes that decreases the current output under light‐off conditions. Therefore, the defect states are strong units to generate or recombine electrons and holes.

**Figure 2:**
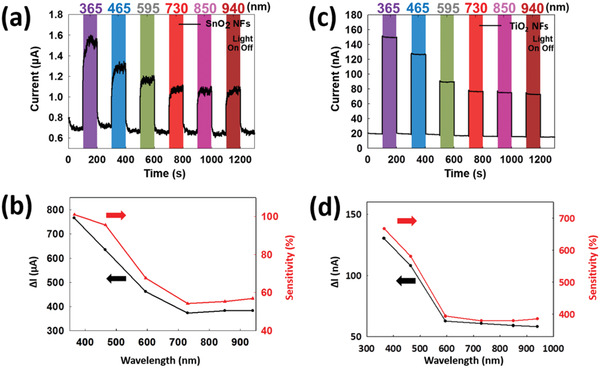
The multiple‐wavelength light‐sensing properties, current variation, and sensitivity of SnO_2_ and TiO_2_ PNFDs. a) The multiple‐wavelength light (365, 465, 595, 730, 850, and 940 nm)‐sensing properties of the SnO_2_ PNFD. b) The current variation Δ*I* (black arrow) and sensitivity *S* (red arrow) of the SnO_2_ PNFD. c) The multiple‐wavelength light (365, 465, 595, 730, 850, and 940 nm)‐sensing properties of the TiO_2_ PNFD. d) The current variation Δ*I* (black arrow) and sensitivity *S* (red arrow) of the TiO_2_ PNFD. The SnO_2_ device was fabricated with nanofiber arrays. The TiO_2_ device was fabricated with a single nanofiber.

**Figure 3:**
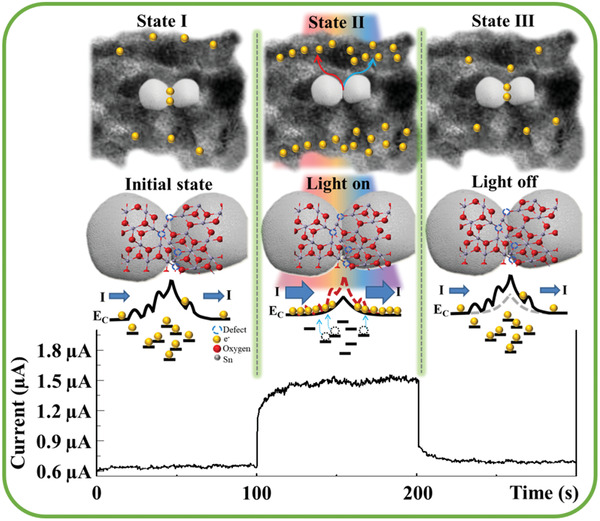
Schematic illustration of the sensing mechanism. State I (initial state): During the fabrication process, the nanofiber has several grain boundaries and interfaces. These grain boundaries and interfaces have numerous defects that trap electrons and lead to low dark current output. State II (illumination state): These defects serve as generation states that generate photocurrent under illumination. State III (non‐illumination state): When illumination ceases, the interface defects convert into recombination centers, which recombine electrons and holes. Hence, instant response and recovery can be achieved by our PNFD.

This PNFD has several advantages, including low initial current and numerous generation‐recombination centers. Initially, the photosensitivity is strongly related to the initial dark current (*I*_D_). The sensitivity can be calculated as follows

(1) $$\begin{array}{c} \text{S}=\frac{I_{\text{P}}-I_{\text{D}}}{I_{\text{D}}}\times 100\% \end{array}$$

where *S* is the sensitivity, *I*_P_ is the photocurrent, and *I*_D_ is the initial dark current. The PNFD delivers a low dark current owing to the abundant grain boundaries and interfaces. Second, the grain boundary and interface have numerous defect states that act as generation centers that generate high photocurrent (*I*_P_). Based on the above two properties, the sensitivity of PNFDs is higher when the device has a low *I*_D_, as shown in Figure 2b,d. The abundant interfaces and grain boundaries also have other benefits, such as a fast response time. As for the generation states, the photo electron–hole pairs are generated from the different energy levels of defects and are immediately separated by the electrical field to form the photocurrent. The generation centers become the recombination centers, which recombine the electron–hole pairs. Thus, instant response and recovery are achieved using our PNFDs. The photocurrent generation is no longer limited to UV light detection and the photocatalytic ability can be enhanced owing to the variety of light sources that can be used. In our previous study, the interface defects between the Pt, Ni, and Si ternary NW heterostructure interfaces exhibited a photoresponse with infrared light (940 nm).^[^ ^53^ ^]^ The advantages of our PNFDs are their high sensitivity, fast response, and UV–visible light photocatalysis.

According to the WHO, over 3.8 million people, including children, are exposed to HAP environments worldwide. The most important HAP issue is toxic gas. CO and NO toxic gasses cannot be detected by humans. Several research groups have reported the relationship between blood carboxyhemoglobin (COHb) levels and CO concentration. Goldstein, and Köthe and Radke conducted studies to determine the effects of exposure to different CO concentration (35–1600 ppm). Their results indicated symptoms including headaches within 6–8 h (35 ppm, COHb < 10%), nominal headaches within 1–2 h (100–200 ppm, COHb ≈ 0–20%), frontal headaches within 1–2 h (400 ppm, COHb ≈ 30–40%), convulsions within 45 min (800 ppm, COHb ≈ 40–50%), and death in less than 2 h (1600 ppm COHb ≈ 60–70%).^[^ ^5^ ^]^ PNFDs have many advantages, including numerous grain boundaries and interface defects between the nanograins that provide numerous random defect states for absorption/desorption units for gas detection. The SnO_2_ PNFD could be used as a CO sensor to avoid the HAP threat, as shown in **Figure** **4**a.

**Figure 4:**
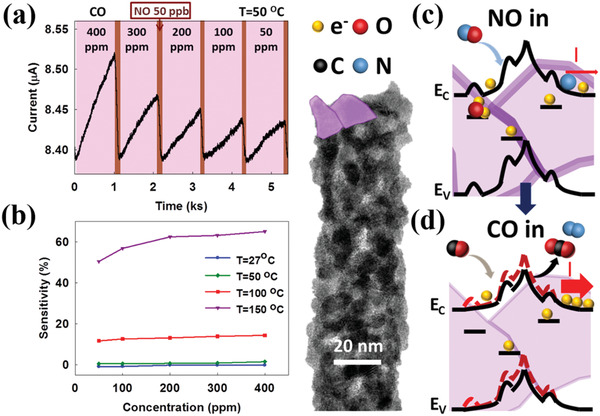
The gas detection ability. CO and NO were used as reducing and oxidizing gases, respectively. a) The detection ability of the alternated different concentrations of CO and 50 ppb of NO with a temperature of 50 °C. b) The sensitivity comparison under different operating temperatures. The gas response was significant at 100 °C and the sensitivity enhancement increased as the operating temperature was increased to 150 °C. The detection mechanisms can be seen in (c,d). When NO had been absorbed by the defects, the interface potential increased to reduce the current flow. Conversely, the CO absorption reduced the surface potential to release the trapped electrons and increased the current.

The SnO_2_ PNFD can operate at a low temperature (50 °C) and detect CO concentrations as low as 50 ppm. The different temperature and concentration detection abilities are shown in Figure 4b. The PNFD also functioned at an operating temperature of over 100 °C. In addition, the sensitivity significantly increased when the operating temperature was increased to 150 °C. The polycrystalline nanofibers had a greater response than other single‐crystalline nanostructures because of the large number of defects resulting in increasing possibilities for gas absorption. The proposed detection mechanism is shown in Figure 4c,d. The absorption of oxidizing gases could increase the potential of the interface and reduce the current output. When the reducing gases (CO) flow into the system, they accelerate the oxidizing gas desorption and increase the current output, which further increases the gas‐sensing response. The PNFD had an extraordinary detection ability for the oxidizing gas, NO. NO is a gaseous signaling molecule involved in many biological and biomedical processes.^[^ ^54^, ^55^ ^]^ Gas‐sensing abilities can be represented by two key parameters, namely, sensitivity and response time of gas detection. Using the PNFD mentioned in this research, the detection concentration and response time of NO at 50 °C was 50 ppb and 40 s, respectively. The recovery process takes 1 ks, as shown in Figure 4a. This property is not beneficial for the commercialization of PNFDs.

Current in the PNFD was generated using a light‐emitting diode (LED) light source. A design was proposed to reduce the reaction time using photo‐assisted methods. The sensitive response to NO and fast recovery after detection are shown in **Figure** **5**a. Various NO concentrations (400–50 ppb) could be detected, and the recovery time was considerably enhanced to 252–40 s. The recovery time of NO detection was improved from 1 ks to 40 s owing to the photo‐assisted property of the PNFDs. Because gas detection was mainly under thermal dynamic control, the detection abilities were enhanced with the increase of the operating temperature, as illustrated in Figure 5b.

**Figure 5:**
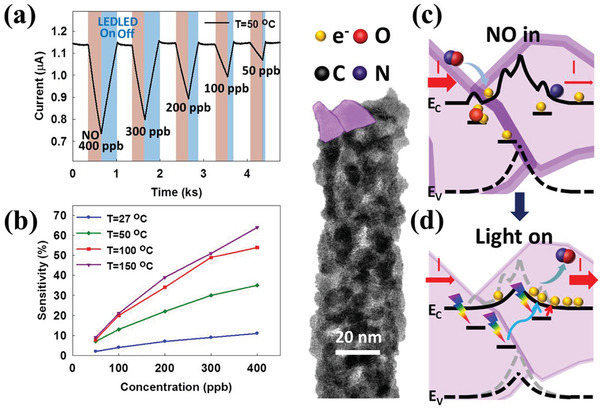
The low‐temperature detection ability of various NO concentrations. a) The sensitivity of PNFDs to various NO concentrations. Detection was achieved from 400 to 50 ppb under an operating temperature of 50 °C. b) The sensitivity of PNFDs at various operating temperatures. Detection could be achieved at low temperatures. The mechanisms of fast gas response for photo‐assisted gas sensors are shown in (c,d). The generation of electron–hole pairs accelerates desorption of NO and facilitates a faster recovery time under 465 nm light illumination.

This photon‐assisted gas detection mechanism improved the sensitivity and recovery time of PNFDs, which benefited from the advantages of the polycrystalline structure. The polycrystalline structure materials provided more surface defects and defect energy levels between nanograins. Surface defects, such as oxygen vacancies (Vo^++^), could drastically increase the probability of gas reaction on the grain surface and interfaces. Additionally, multiple defect energy levels could enhance the abilities of photocurrent generation, as shown in Figure 5c,d. LED light illumination at 465 nm generated electron–hole pairs to accelerate the NO desorption and shorten the recovery time. With the UV–visible photoelectric effect and strong gas detection ability, these PNFDs could be potential candidates for household applications.

Although the WHO has warned people to reduce the burning of solid fuels, liquid fuels, and incense, attempts to reduce air pollution need to be increased. The burning of incense is of special importance in Asian countries, due to the mainstream religions of Taoism and Buddhism. According to Lin et al.,^[^ ^56^ ^]^ the clinical, structural, and molecular effects of incense smoke show that it contains PM, gas products (such as CO, CO_2_, NO_2_, and SO_2_), and many organic compounds. When people are exposed to an organic PM environment for long periods, the PM enters the body through the nose and mouth, which results in diseases such as chronic obstructive pulmonary disease, pneumonia, ischemic heart disease, lung cancer, and may lead to death. The PM pollution issue inspired us to think about how to solve and prevent the threat from PM and microdust at homes.

Hence, a Sn‐PVA SnO_2_ polymer nanofiber film with strong surface static forces and a porous structure was fabricated to act as a PM air filter. We generated PMs and microdust by burning incense and allowed the incense smoke to pass through the polymer nanofiber air filter (without any pressure). Microdust and PMs were easily captured using the Sn‐PVA SnO_2_ polymer nanofibers as opposed to nonwoven filters, as shown in the SEM images in Figure S3a,b, Supporting Information. The PMs capture process was also recorded by in situ optical microscopy, as shown in Figure S3c–e and Video S1, Supporting Information. The strong surface static Sn‐PVA SnO_2_ polymer nanofiber can be used as a PM air filter, with many advantageous properties, including strong electrostatic force, and high PMs and microdust removable properties, to negate the threat from PMs and microdust for people.

In this work, we provided defect‐rich polycrystalline SnO_2_ and TiO_2_ nanofibers, which have great potential for the photoelectric effect by triggering by UV–visible light (365, 465, 595, 730, 850, and 940 nm). Owing to the strong nanoeffects, such as surface/interface defects and static force, the toxic gases (CO and NO) and PMs can be easily sensed and captured. A low operating temperature (50 °C) and fast recovery time can lead to next‐generation commercial household appliances to monitor indoor air pollution. The UV–visible light photoelectric effect and PMs‐capturing properties can also be powerful parameters for negating air pollution (PMs, microdust, and bacteria). With the strong nanoeffects and photoelectric effect of defect‐rich SnO_2_ and TiO_2_ PNFDs, the CO and NO gas sensor, UV–visible photoelectric nanofilm, nano‐photocatalyst PM filters, air conditioners, and intelligent nano‐photocatalyst toxic gas air cleaners can be achieved. Defect‐rich SnO_2_ and TiO_2_ PNFDs provide an intelligent way to control and reduce indoor air pollution.

## Experimental Section

##### Fabrication of Polycrystalline SnO_2_ and TiO_2_ Nanofibers

The Sn‐PVA precursor solution for the electrospinning system was used to fabricate SnO_2_ polycrystalline nanofibers with the addition of SnCl_4_∙5H_2_O and a polymer solution containing 15 wt% of polyvinyl alcohol (PVA) in deionized water (DI) under vigorous stirring. The electrospinning system was composed of a Sn‐PVA precursor‐loaded syringe, a needle tip, a high‐voltage power supply, and a ground collector. The Sn‐PVA polymer nanofibers were annealed in a furnace to obtain SnO_2_ polycrystalline nanofibers. The Ti‐PVP precursor solution for the electrospinning system was used to fabricate TiO_2_ polycrystalline nanofibers with the addition of titanium(IV) isopropoxide and a polymer solution containing 9 wt% of polyvinyl alcohol (PVA) in DI water under vigorous stirring. The electrospinning system consisted of a Ti‐PVP precursor‐loaded syringe, a needle tip, a high‐voltage power supply, and a ground collector. The Ti‐PVP polymer nanofibers were annealed in a furnace to obtain TiO_2_ polycrystalline nanofibers.

##### Fabrication of Multiple‐Wavelength Photon‐Sensing Devices

The SnO_2_ and TiO_2_ polycrystalline nanofibers were placed on an indium tin oxide (ITO) electrode glass substrate. Silver paste was used to connect the nanofibers and ITO electrodes on both ends to form an ohmic contact device.

##### Characterization and Measurements

High‐resolution images of multiporous and nanograin structures of nanofibers were obtained using a transmission electron microscope (JEOL JEM‐2100F‐HR). A scanning electron microscope (ZEISS SIGMA FE‐SEM) was used to analyze the surface nanostructure and rough interface of the nanofibers in high‐resolution images. The operating bias of the current–time (*I*–*T*) measurement was conducted under 1 V. The electrical measurement and analysis system were connected to a chamber with electrical probes, CO_(g)_ and NO_(g)_ gas flow system, pump, and LED light source.

## Conflict of Interest

The authors declare no conflict of interest.

## Supporting information

Supporting Information

Supplemental Video 1
