# Supplementary material for: Enhancement in the Detection Ability of Metal Oxide Sensors Using Defect‐Rich Polycrystalline Nanofiber Devices
Source: Glob Chall. 2020 Sep 28;4(11):2000041. doi: 10.1002/gch2.202000041 (PMC7607247; doi:10.1002/gch2.202000041)
Supplement: Supplementary file 1 — Supporting Information [file GCH2-4-2000041-s001.pdf]

© 2020 Wiley-VCH GmbH

Global Challenges

Supporting Information

for *Global Challenges,* DOI: 10.1002/gch2.202000041

Enhancement in the Detection Ability of Metal Oxide Sensors Using Defect-Rich Polycrystalline Nanofiber Devices

Chun-Yen Lai, Yu-Ting Lin, Hung-Kun Hsu, Ding-Yeong Wang, Wen-Wei Wu, and Ping-Hung Yeh**

Copyright Wiley-VCH GmbH, Germany, 2020.

Supporting Information

Enhancement in the Detection Ability of Metal Oxide Sensors Using Defect-rich Polycrystalline Nanofiber Devices

Chun-Yen Lai[a], *Yu-Ting Lin*[d], *Hung-Kun Hsu*[d], *Ding-Yeong Wang*[e], *Wen-Wei Wu*[a,b,c,*], *and Ping-Hung Yeh*[d,*]

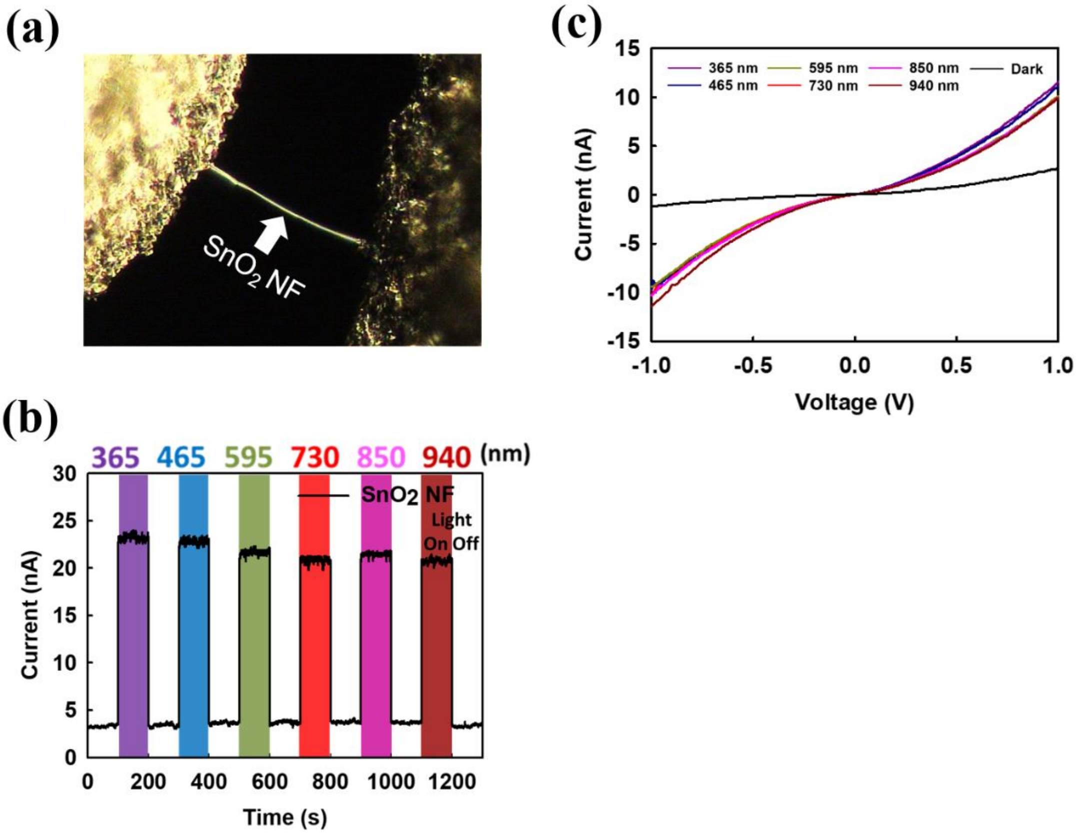

Figure S1. **The broadband light sensing properties of single SnO_2 PNFD.** (a) The OM image of a single SnO_2 PNFD. (b) The multiple wavelength light (365, 465, 595, 730, 850 and 940 nm) sensing properties of an SnO_2 PNFD measured in an ambient environment. (c) The I-V curve of a single SnO_2 PNFD with multiple wavelengths (365, 465, 595, 730, 850 and 940 nm).

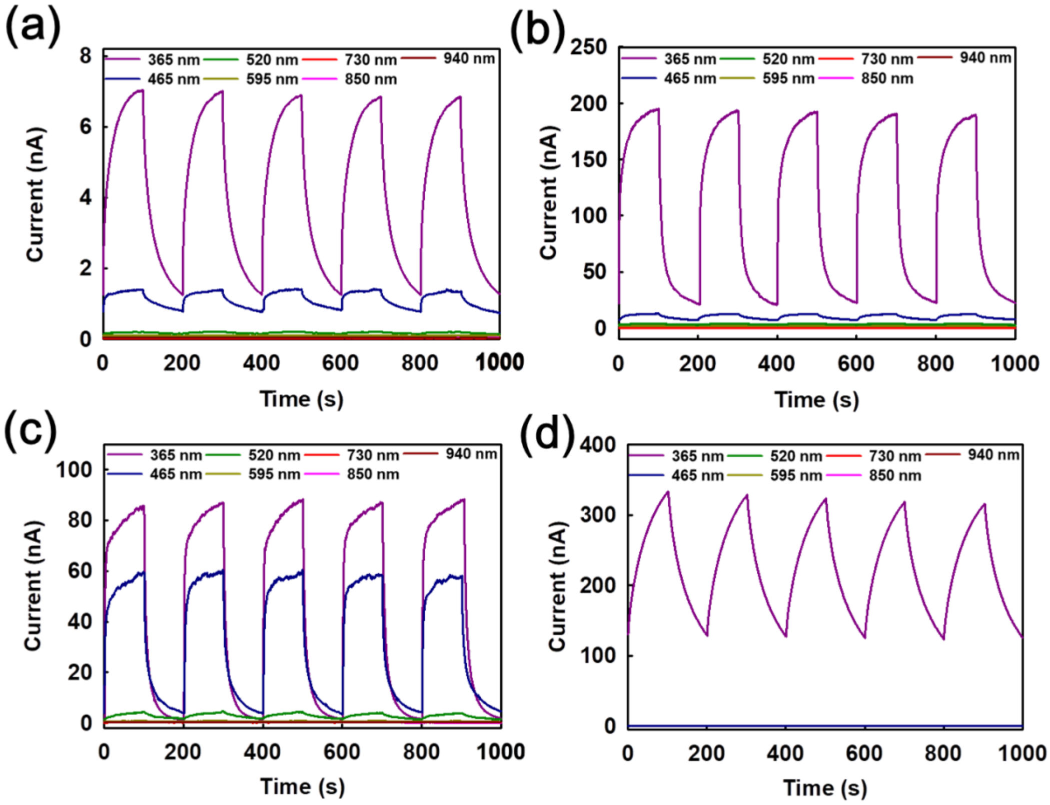

Figure S2. The multiple wavelength light sensing properties of a TiO_2 PNFD with different hydrogen plasma treatments. (a) Without hydrogen plasma treatment. (b) With hydrogen plasma treatment at 40 W for 30 s. (c) With hydrogen plasma treatment at 80 W for 30 s. (d) With hydrogen plasma treatment at 120 W for 30 s.

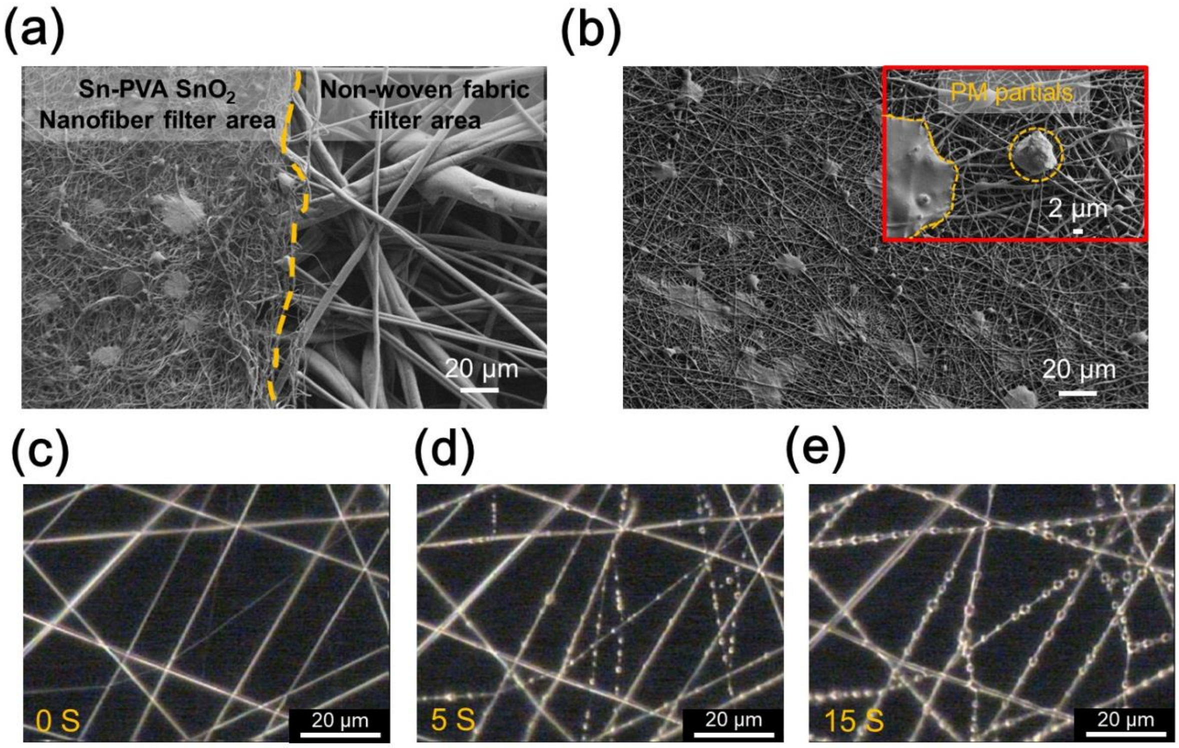

Figure S3. **The micro dust and PM removal performance of Sn-PVA SnO_2 polymer nanofiber filters.** (a-b) SEM images of a Sn-PVA SnO_2 polymer nanofiber filter and a non-woven fabric filter with captured micro dust and PM. (c-e) *In-situ* optical microscope images of the process of micro dust and PM capture at different times.
